# Hemosuccus Pancreaticus on Endoscopy

**DOI:** 10.14309/crj.0000000000000814

**Published:** 2022-09-14

**Authors:** Sean Lee, Michael Gavin

**Affiliations:** 1 Department of Medicine, University of Arizona College of Medicine, Tucson, AZ; 2 Division of Gastroenterology and Hepatology, Department of Medicine, Southern Arizona Veterans Affairs Health Care System, Tucson, AZ

## CASE REPORT

**Figure 1.**
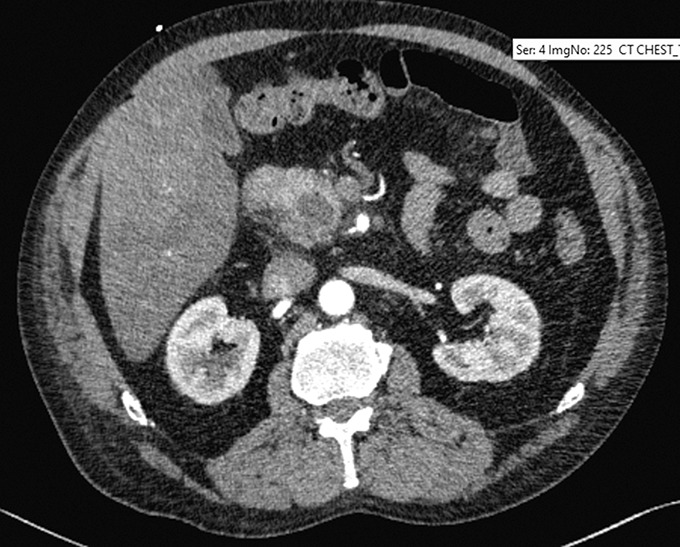
Computed tomography abdomen showing peripancreatic fat stranding with a hypodense area in the pancreatic head/uncinate.

**Figure 2.**
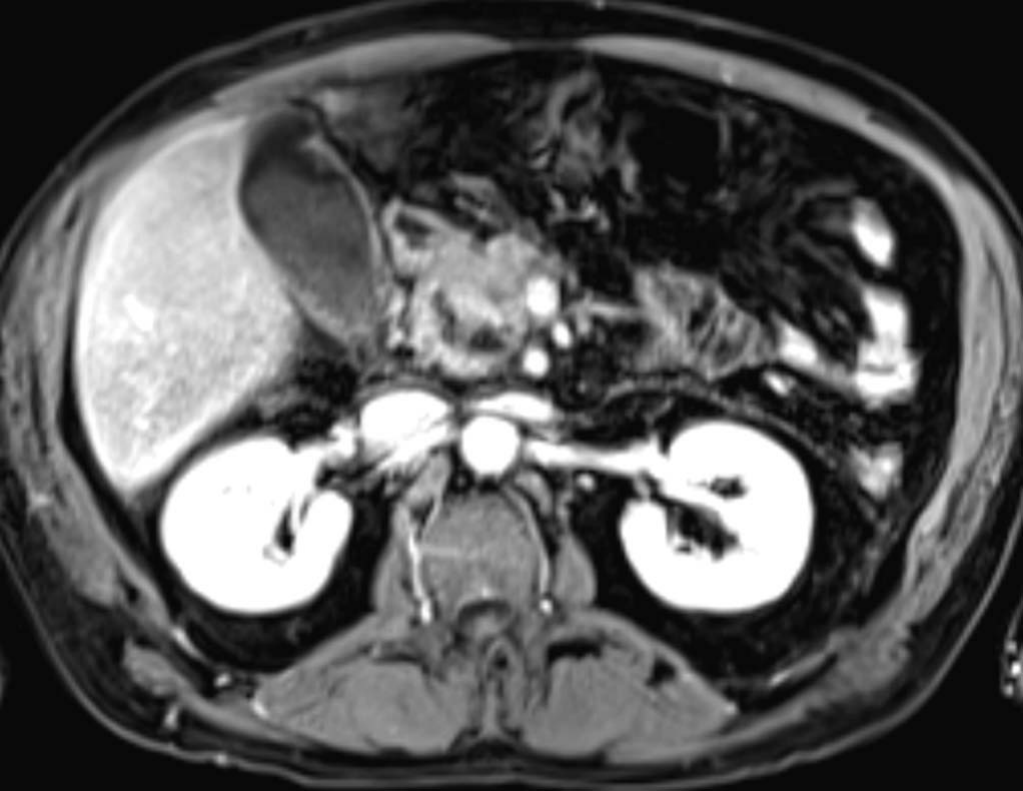
Magnetic resonance imaging of abdomen showing acute pancreatitis with necrotic collections containing fluid, debris, hemorrhage.

**Figure 3.**
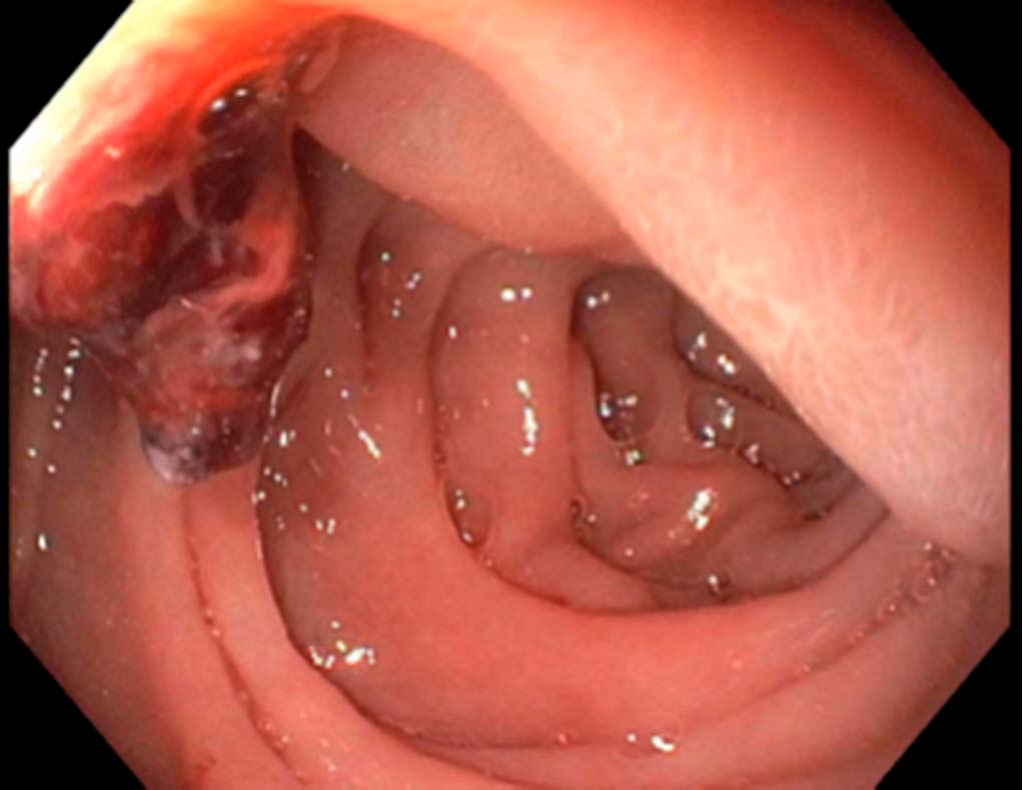
Endoscopy revealing blood clot protruding from ampulla.

**Figure 4.**
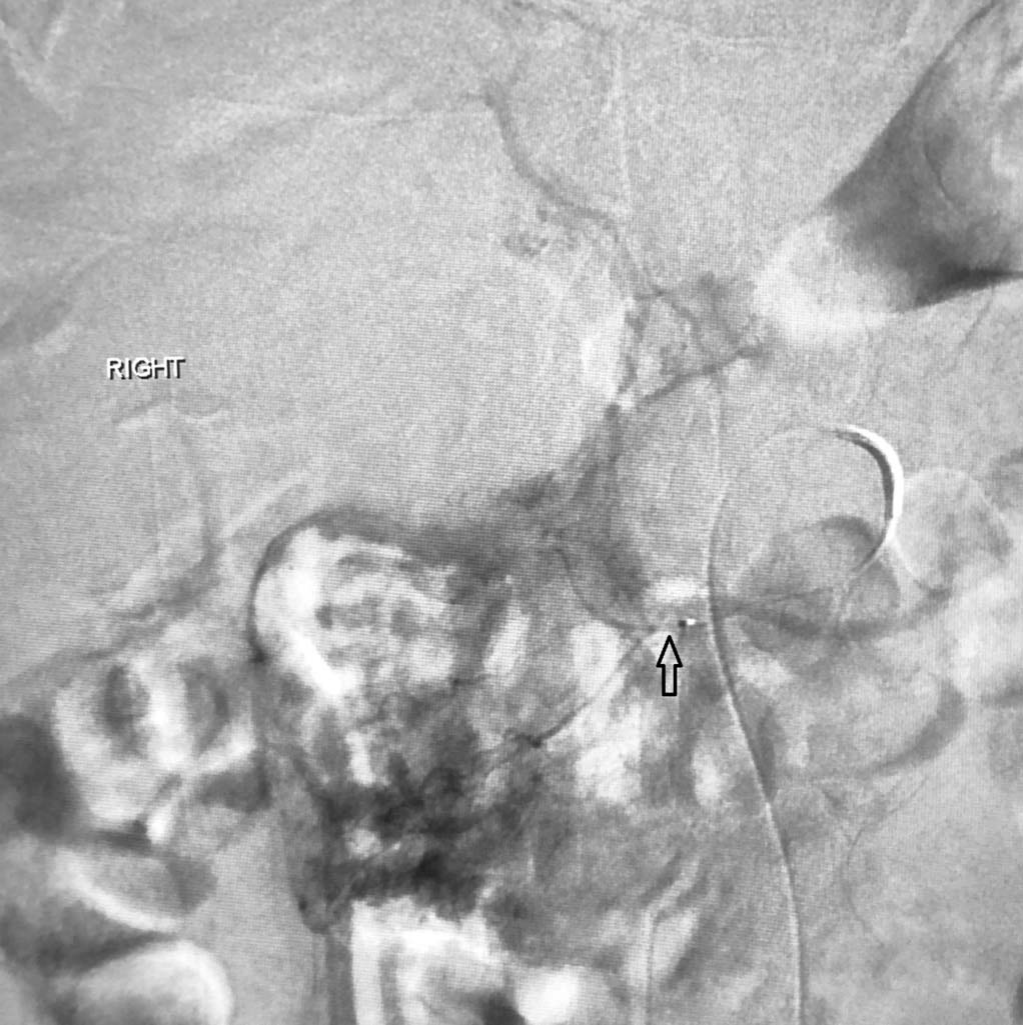
Angiography showing eroded gastroduodenal artery.

A 55-year-old man with chronic alcohol use presented with epigastric pain radiating backward, vomiting, and 1 day of decreased colostomy output (created 1 year earlier for an infected hernia-repair mesh). He had no history of pancreatitis. Computed tomography angiography of the abdomen revealed pancreatic fat stranding, peripancreatic head fluid collection, and a portal vein thrombus (Figure 1). Heparin drip was started. On day 4, the patient developed maroon stools in the colostomy bag, requiring transfusion of 2 units of red blood cells. A magnetic resonance cholangiopancreatography found a 3.1 and a 2.6 cm necrotic fluid collection filled with debris and hemorrhage at the pancreatic head (Figure 2). An emergent esophagogastroduodenoscopy revealed a blood clot protruding from the ampulla (Figure 3). Next, interventional radiology performed a same-day visceral angiography; they discovered and coiled an eroded gastroduodenal artery and pancreaticoduodenal arcades of the superior mesenteric artery (Figure 4). This resolved the patient's bleeding. Roughly 20% of moderately severe acute pancreatitis cases have local complications (eg, pseudocysts, necrosis, and hemosuccus pancreaticus).^1^ Hemosuccus pancreaticus is a rare cause of upper gastrointestinal bleeding, estimated at 1/1,500 cases, translating to difficult/delayed diagnosis and high mortality (overall estimated at 9.6%; 90% if untreated).^2^ It is defined as bleeding into the pancreatic duct and often related to inflammatory pancreatic diseases, pancreatic pseudocysts, and pancreatolithiasis.^3^ Workup with imaging such as magnetic resonance cholangiopancreatography or the gold-standard computed tomography angiography should be pursued when it is suspected. Endoscopy may make the diagnosis in up to 64% of patients and should be performed to rule out other causes of bleeding.^4^ Intermittent bleeding renders visualization difficult, although side-viewing endoscopy may significantly enhance diagnostic yields.^4^ Endoscopic ultrasound and endoscopic retrograde cholangiopancreatography may also aid in detecting filling defects of the pancreatic duct.^4^ Visceral angiography successfully treats over 79% of cases, although severe cases may require pancreaticoduodenectomy.^5^

## DISCLOSURES

Author contribution: S.L.—drafting and critical revisions to the article, literature review, provided images, and is the guarantor. M.G.—critical revisions and approval of the article.

Financial disclosure: None to report.

No Informed Consent was obtained for this report.

## References

[1] Banks PA Bollen TL Dervenis C . Acute pancreatitis classification working group. Classification of acute pancreatitis--2012: Revision of the atlanta classification and definitions by international consensus. Gut. 2013;62(1):102–11. 23100216 10.1136/gutjnl-2012-302779

[2] Yu P Gong J. Hemosuccus pancreaticus: A mini-review. Ann Med Surg (Lond). 2018;28:45–8. 29744052 10.1016/j.amsu.2018.03.002 PMC5938526

[3] Dinu F Devière J Van Gossum A The wirsungorrhagies: Causes and management in 14 patients. Endoscopy. 1998;30:595–600. 9826136 10.1055/s-2007-1001362

[4] Yashavanth HS Jagtap N Singh JR Hemosuccus pancreaticus: A systematic approach. J Gastroenterol Hepatol. 2021;36:2101–6. 33445212 10.1111/jgh.15404

[5] Rammohan A Palaniappan R Ramaswami S Hemosuccus pancreaticus: 15-year experience from a tertiary care GI bleed centre. ISRN Radiology. 2013:191794. 24959558 10.5402/2013/191794 PMC4045512

